# Cervical cancer screening program based on primary DNA-HPV testing in a Brazilian city: a cost-effectiveness study protocol

**DOI:** 10.1186/s12889-020-08688-4

**Published:** 2020-04-28

**Authors:** Julio Cesar Teixeira, Diama Bhadra Vale, Joana Froes Bragança, Cirbia Silva Campos, Michelle Garcia Discacciati, Luiz Carlos Zeferino

**Affiliations:** 1 Department of Obstetrics and Gynecology, University of Campinas (UNICAMP), Rua Alexander Fleming, 101 – Cidade Universitaria, Campinas, SP 13083-881 Brazil; 2 Division of Gynecologic and Breast Oncology, Women’s Hospital, UNICAMP, Rua Alexander Fleming, 101 – Cidade Universitaria, Campinas, SP 13083-881 Brazil

**Keywords:** Cervical cancer, Papillomavirus infections, Public health, Cancer screening, Pap smear, HPV DNA test

## Abstract

**Background:**

The causal relationship between high-risk (hr) HPV infection and precancerous lesions or cervical cancer has led to the development of strategies to increase screening performance and prevent this cancer. The increased sensitivity of DNA-HPV testing compared to cervical cytology favors DNA-HPV testing as a primary screening test. Cervical cancer screening in Brazil is opportunistic, and this cancer remains a considerable health problem with a high proportion of diagnoses in advanced stages. This paper aims to describe the design and implementation of the Cervical Cancer Screening Program with primary DNA-HPV testing (CCSP-HPV) planned for Indaiatuba City (SP), Brazil; the strategies to achieve higher population coverage; and a study protocol for cost-effectiveness analyses.

**Methods:**

The CCSP-HPV was designed based on successful guidelines that replaced cervical cytology-based screening by the DNA-HPV test performed at 5-year intervals. The screening will be performed for the female population aged 25-64 years cared for by the public health system and aim to reach 80% coverage after completing the first round. The chosen DNA-HPV test detects 14 hr-HPV types and genotypes HPV-16 and 18. All women with a negative test will be reassessed after five years. Women showing a positive test for HPV-16 and/or 18 will be referred for colposcopy. Those showing the other 12 hr-HPV types will be tested by cytology, and if any abnormality is detected, they will also be referred for colposcopy. The histopathologic evaluation will be reviewed by a pathologist panel and aided by p16 immunohistochemistry. A cost-effectiveness analysis will be performed by a Markov model comparing the cost of the new program and the screening performed by conventional cytology five years prior (2011–2016).

**Discussion:**

The new screening program is considered a breakthrough for public health regarding cervical cancer, which is the third leading cause of cancer death among Brazilian women. Achieving at least 80% coverage will have the possibility to change this scenario. The proposed program will provide a modern cervical cancer screening method for women, and information about cost-effectiveness will help other similar places support the decision of implementing cervical cancer screening using the DNA-HPV test.

## Background

Cervical cancer remains a major health problem, especially in developing countries such as Brazil [1, 2]. The widespread knowledge that cervical cancer is caused by persistent high-risk human papillomavirus (hr-HPV) infection has resulted in the development of biomolecular tests and alternative strategies for cervical preneoplastic lesion screening. Cytology-based cervical cancer screening (e.g. Papanicolaou test), colposcopy and histological diagnosis followed by the treatment of cervical intraepithelial neoplasms (CINs) have been widely used worldwide. Cervical cancer mortality reduction has been observed in high income and some middle-income countries, especially in those that have established successfully organized cancer prevention programs [3, 4].

However, in low- and middle-income countries, the success of screening is hampered by many factors, such as low cytology sensitivity, requiring frequent repetition to achieve longitudinal sensitivity, low coverage of the target population at regular intervals, and poor quality control of cytology services [3, 4]. These factors promote the search for alternative strategies, such as HPV molecular testing.

Several randomized clinical trials and prospective studies from the early 2000s to the present have shown the superiority of DNA-HPV testing to detect high-grade squamous intraepithelial lesions (HSILs), adenocarcinoma in situ (AIS) and early cervical cancer when compared to cervical cytology [5–9]. A meta-analysis study that included four European clinical trials concluded that screening with the DNA-HPV test may be conducted in women aged 30 years and older in at least five-year intervals [10].

The ATHENA study evaluated the Cobas® HPV test (Roche Molecular Systems, Pleasanton, CA, USA) as a primary screening test for cervical cancer in over 42,209 women, and the findings support the use of a combination of HPV-16/18 genotyping and reflex cytology only for the group of other hr-HPV positive, non-HPV-16/18 women for colposcopy triage. HPV-16/18 genotyping stratifies a high-risk group for HSIL/AIS, increasing the specificity of the test and making it possible to use it for screening starting at age 25 years, avoiding the implementation of hybrid strategies that use cervical cytology for younger women [9].

The DNA-HPV test has a negative predictive value of approximately 100% and allows a screening interval of at least five years, longer than the 3-year interval for cytology. These would reduce the total number of tests performed, affecting costs. Some studies have provided strong evidence for the adoption of the DNA-HPV test in cervical cancer screening [11–14].

The Food and Drug Administration (FDA), in April 2014, approved the DNA-HPV testing that includes genotypes 16 and 18 (Cobas® HPV) for primary screening of cervical cancer [15]. More recently, the Society of Gynecological Oncology and the American Society of Colposcopy and Cervical Pathology have proposed a guideline for cervical cancer screening based solely on the use of DNA-HPV tests for women over 25 years of age [16]. Australia also approved the primary screening of HPV, with colposcopic evaluation for HPV 16/18 and reflex cytology for other types of HPV, starting at 25 years of age and at intervals of five years when the result is negative for HPV [17].

Cervical cancer is the third most common cancer among women in Brazil, with an estimated 16,350 new cases in 2018 [18]. Current recommendations from the Brazilian Ministry of Health state that cervical cancer screening is based on cervical cytology, which should be repeated every 3 years after two consecutive annual negative examinations, in women aged 25-64 years [19]. Data show an excess of cytology due to the opportunistic screening currently practiced in Brazil, with approximately 50% of cervical cytology performed annually and only 10% within a three-year interval recommended by the Brazilian protocols. Additionally, approximately 20% of screening tests are performed on women under 25 years of age [20, 21]. Besides, there is a large proportion of cases diagnosed at advanced stages, most of them managed at Public Health System [21, 22]. These factors highlight the need for reassessment of the cervical cancer screening program currently in place in Brazil.

In 2017, the city of Indaiatuba, in São Paulo State, was the pioneer in Brazil deciding to replace cervical cytology with the primary DNA-HPV test for primary cervical cancer screening. This paper aims to describe the implementation of the *Cervical Cancer Screening Program with Primary DNA-HPV testing (CCSP-HPV)*, the strategies designed to reach higher population coverage, and a study protocol for the cost-effectiveness analyses.

## Methods/design

### Design, study setting and study population

This is a longitudinal study involving women from Indaiatuba (Sao Paulo State, Brazil), an urban city with a population of 240,000 people and a high (0.79) human development index [23]. The target women are women relying only on public health services (SUS – Unified Health System) and candidates for cervical cancer screening. Cervical cancer screening in Brazil includes women aged 25-64 years.

#### Sample size calculation

The female population 25-64 years old comprises 63,362 subjects for Indaiatuba, according to the official Brazilian Census (2010) and estimates for 2014 [24]. In Brazil, health care is free of charge to every citizen, although some people co-use private services. In Indaiatuba, around 50% of the female population uses private services, and the other half relies exclusively on the SUS to access health care. Therefore, the target SUS population was 31,681women for the new program. As the goal of the program is to reach at least 80% coverage of the target population after five years, the population of the study was estimated to be 25,000 women in five years, which means at least 5000 women tested per year.

The organization of the CCSP-HPV implies developing strategies to reach higher target population coverage, evaluate the compliance of the staff to the program, and research to evaluate the cost-effectiveness. For comparison, a similar population from the same city that performed routine cytology screening from 2012 to 2016, five years before CCSP-HPV will be the reference for comparison of cost-effectiveness evaluation. This reference population (years 2012–2016) performed nine to eleven thousand pap tests per year (total 54,000 to 66,000 tests), but with a low agreement, about 33%, with the official recommendations for pap screening.

### Eligibility

Inclusion criteria for CCSP-HPV

- women have started sexual activity aged 25 to 64 years are the target for regular screening test (women over 64 years will be permitted for a single exit test by personal request);
- women physically well enough to undergo a pelvic exam.

The inclusion criteria evaluation should be postponed if any of the following conditions are met: 40 days postpartum period, abundant genital bleeding, cervicovaginal infection and having underwent the last cervical cytology in the last 12 months.

Exclusion criteria

- women who have already undergone DNA-HPV testing for different medical purposes except screening;
- women with a previous diagnosis of the cervical lesion followed in specialized clinics;
- women who underwent a total hysterectomy.

Control group:

- All women from Indaiatuba that underwent cytology tests by SUS from 2012 to 2016. These women will be identified from the regional central laboratory database, located at the Cytopathology Laboratory of Women’s Hospital (University of Campinas - UNICAMP). All information about the compliance of the staff to follow the official recommendations related to conducting the women with abnormal pap, as the follow-up and/or treatment, are available at Colposcopy Unit in Indaiatuba City and Women’s Hospital (UNICAMP).

### Organization of the screening with primary DNA-HPV test and study procedures

#### HPV test selection

It should be noted that approximately 70% of invasive squamous carcinomas and 82% of adenocarcinomas are related to HPV-16 and/or 18 [25]. A study that analyzed data from more than 20,000 women for ten years showed the higher cumulative incidence of CIN3 in those women with initial infection of HPV-16 and/or 18 than in those infected with 11 other types of hr-HPV [26]. The availability of genotyping HPV-16 and 18 made it possible to evaluate the test performance for screening women aged 25 to 29 years and maintaining the same target age as the official Brazilian program. Considering these factors, the FDA-approved Cobas® HPV Test (Roche Molecular Systems, Pleasanton, CA) was defined as the HPV test for CCSP-HPV. This HPV test provides individual results on the highest risk genotypes - HPV 16 and HPV 18 - and aggregated results on the twelve other hr-HPV genotypes (types 31, 33, 35, 39, 45, 51, 52, 56, 58, 59, 66 and 68).

**Fig. 1:**
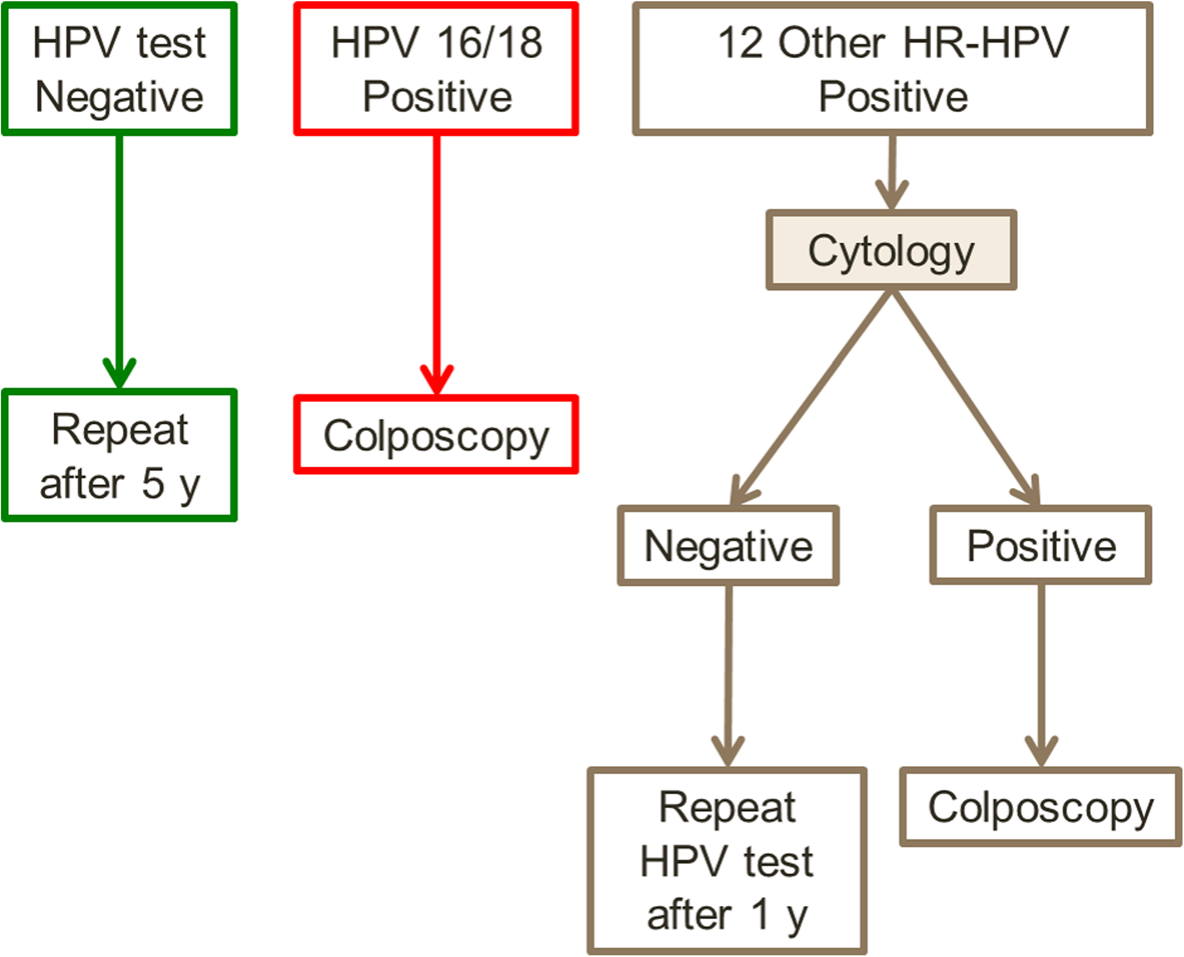
Overview of cervical cancer screening guidelines using a primary DNA-HPV test with HPV-16 and 18 genotyping in Indaiatuba City (SP), Brazil

In addition to the DNA-HPV test, the sample vial will be stored for cytology evaluation, if necessary and according to the CCSP-HPV management flowchart (Fig. 1), preventing the women having to return for another sample collection.

For this study, the Cobas® HPV Test will be performed in a central laboratory, the Laboratory of Women’s Hospital (HPV Test Lab) located at the University of Campinas (UNICAMP), Campinas (SP), 45 km away from Indaiatuba. The Cobas® HPV Test runs on the advanced Cobas® 4800 System (Roche Molecular Systems, Pleasanton, CA, USA).

#### Defining the management flowchart

CCSP-HPV will last five years. The tests and laboratory will be the same for the entire five-year period planned for the study. Figure 1 represents the flowchart guiding CCSP-HPV. Briefly, the procedures are as follows:

1. Women with negative DNA-HPV tests will be instructed to return after five years to repeat the test.
2. Women who test positive for HPV-16 and/or 18 will be referred for colposcopy.
3. Women positive for the other 12 h-HPV types will also be evaluated by cervical cytology performed in the same sample. In the case of negative cervical cytology, women will return after 12 months to repeat the DNA-HPV test. If any abnormality was detected in cytology (ASC-US, LSIL, HSIL, suspicious for glandular lesion or cancer), the woman will be referred for colposcopy.

#### Cervical sample collection

The collection of cervical samples will be performed in the routine of care at 18 primary health care units in Indaiatuba. The cervical sample will be collected using a brush, and the sample will be stored in a previously identified vial containing preservative medium specific for the HPV test (PreservCyt, ThinPrep – Hologic, Inc., Marbourough, MA, USA). All procedures related to sample collection, storage, and transportation to the HPV testing laboratory are similar to those used in previous cytology screening, except for sample slides (conventional cytology) replaced for liquid-based cytology (LBC) vials.

All primary health care units will be informed about CCSP-HPV guidelines on the collection, storage, and transportation of samples, which will be primarily sent to a central unit in the city and from there to the HPV testing laboratory. This sample transport is planned to take place within one week. The samples can be well kept for up to 6 weeks at room temperature of 15 to 30 degrees Celsius.

#### Liquid-based cytology

In cases of positive tests for the other 12 hr-HPV types, the cytology evaluation and results will follow the Bethesda System Classification 2014. Considering that cervical cytology has a very variable sensitivity for the detection of HSIL, in this study protocol, the cytologists will have prior information on HPV results (tested positive for other 12 hr-HPV types). The strategy is to increase attention to the smallest morphological details and thus increase the sensitivity of the cytology test.

#### Colposcopy and cervical procedures

The colposcopy evaluation will be performed at a referral unit in Indaiatuba, and the indication will be defined according to the CCSP-HPV flowchart (Fig. 1). The colposcopy will follow the standards preconized for the Brazilian Society of Pathology of Lower Genital Tract and Colposcopy and the International Federation of Cervical Pathology and Colposcopy (IFCPC). The colposcopic images will be described and classified according to the lastest colposcopic terminology [27].

The colposcopy unit has two active colposcopists, and they will be informed about the program and to perform colposcopy in cases with positive HPV test results, with or without previous cytology evaluation, a novelty in the routine of these professionals. Previously, these colposcopists will be undergoing a comprehensive test with 20 to 40 colposcopic cases with illustrations to assess the agreement of colposcopic findings with two additional senior colposcopists at Women’s Hospital (UNICAMP). The training will be repeated until the colposcopists reach a good or excellent level in agreement tests. The evaluation of the cervical canal will be performed whenever indicated, according to current guidelines, consisting of cervical canal cytological samples or excision procedures [28]. The cervical biopsy will be guided for colposcopy using biopsy surgical tweezers to take a tissue sample from the suspected area of cervical epithelium, usually 3 to 5 mm in size. In the absence of colposcopic changes, no biopsy will be performed.

Surgical procedures for excision of the transformation zone (EZT) will be performed for diagnostic purposes in women with or suspected HSIL. EZT will be performed using a loop electrosurgical procedure in the same colposcopy unit. In specific clinical situations, the surgical procedure in the cervix will be performed at City Hospital and, exceptionally, by cold knife conization. The Women’s Hospital (UNICAMP) is the reference for more comprehensive support for cervical cancer cases if it occurs in the current organization.

#### Histopathological evaluation

The histopathological evaluation will be performed on cervical tissue samples obtained by biopsies or excision procedures. The pathology laboratory available to serve the routine of the Indaiatuba Health Department will be responsible for this exam. For quality assurance, all biopsies will be tested for p16 immunohistochemistry in an external laboratory. For discrepant or doubt diagnoses, a senior pathologist from the University of Campinas (UNICAMP) will review and the final histologic diagnosis will be established by consensus among pathologists and according to the WHO classification [29].

#### Follow-up

This study protocol will evaluate one round of HPV (five years). Women with positive tests and forwarded colposcopy will return to routine screening 5-year intervals after a colposcopic evaluation is negative and a 12-month follow-up DNA-HPV test is negative. This process will be repeated yearly until the tests become negative. Management and follow-up of HSIL or cancer diagnosis will be guided by the guidelines from the Brazilian National Cancer Institute [28].

### Endpoints

The endpoints of this study protocol include age compliance of the CCSP-HPV, the coverage rate of the target population, frequency of HPV positivity by age group, including 25-29 years old, number of colposcopies generated, and cervical lesions identified and treated. These data will be included in cost-effectiveness analyses based on appropriate and specific endpoints.

### Data analysis

Women’s information and test results will be entered into a single Excel database for the entire municipality. Some information will also be present in the Integrated Health Informatics System of Indaiatuba (SALUS System), which is a health management system for the entire city. A more detailed information system for monitoring screening population management according to the flowchart proposed for the CCSP-HPV protocol (schedule for sample collection, including follow-up cervical cytology and/or colposcopy, DNA-HPV test results, colposcopy, biopsy, EZT, and test repetition) is in development, which will be integrated into the SALUS System. Information will be discriminated for every primary health care unit. Thus, we will have the opportunity to monitor and identify areas with adequate population coverage and areas that need further action to achieve adequate coverage.

The databases with information generated from this protocol will be revised and analyzed annually and after the study ends. During the five years planned for the study, women inclusion and additional health procedures for those with positive HPV testing will be monitored every 30 days.

A cost-effectiveness survey is planned for this study, and it will be compared with the screening performed by conventional cervical cytology five years prior. Such analysis will also consider the diagnostic rates of precursor lesions and cervical cancer corresponding to the HPV test or cervical cytology-based periods. Indaiatuba public health system and regional health service (Women’s Hospital, UNICAMP) records will be reviewed looking for cases of HSIL, AIS or cervical cancer diagnosed and managed out of the screening set in women from Indaiatuba.

For cost-effectiveness analyses, the Markov model will be built, and the detailed process will be subject to future publication. Cost information regarding the screening process will be collected for the previous cytology screening and during the implementation of the new program.

Qualitative analysis is also foreseen to evaluate the perception of the population and health professionals regarding the modification of the method of cervical cancer screening in the city.

### Ethical aspects

The mayor of Indaiatuba City approved a law in 2017 replacing standard cytology for screening by HPV test in all Public Health Care System and the CCSP-HPV became a standard of care. The Ethics Committee of UNICAMP approved the study protocol (number 1045580, May 1, 2015) to evaluate CCSP-HPV implementation and cost-effectiveness. The Ethics Committee also approved the waiver of the Informed Consent Form, considering that this study will analyze data from health system records, without direct contact with patients.

## Discussion

This study is considered progress in public health and is aligned with the proposals of screening programs in different places and countries worldwide who started implementation since 2014 [16, 30–32], with a considerable perspective for improving health care offered to the population.

The main point is to make it possible to achieve an adequate organization status of cervical cancer screening in the public health care scenario and reach more than 80% coverage over the target population, women 25-64 years old. Another important problem foreseen for the near future that can be addressed is about screening in vaccinated women, what is expected to happen from 2025, when girls vaccinated in 2014 will reach the age to start screening at age 25. There is a tendency for consensus that the DNA-HPV test will be a better way to screen previously vaccinated women [32, 33]. These new actions may change the current scenario regarding cervical cancer in Brazil, where one woman dies every 90 min at a mean age of 45 years.

The number of women never been screened and the real pap screening coverage is unknown at this moment for the majority of Brazilian regions. There is some published date over the metropolitan region where the Indaiatuba City is, which reports an estimates of 15 to 30% pap screening coverage [34], and a high rate of cervical cancer diagnosed in advanced stage [21], suggesting that over two-thirds of the women are out of date or out of the regular screening. Thus, the new screening program proposed for Indaiatuba City will ensure an additional identification of 50% of women who have not had regular preventive exams before. This opportunity will correct a health system distortion that neglects women without periodic preventive tests, and now, starting to use a modern method that requires fewer evaluations in their lives.

The indication of the DNA-HPV test as a primary screening tool for women aged 30 years or more is a consensus (FDA). The CCSP-HPV includes women 25-29 years to save money by employing unique screening technology due to logistical costs and misuse of tests when available [16, 17]. Testing women 25-29 years old was considered a reasonable action because the genotyping available in the Cobas® HPV Test permits the identification of women at higher risk of having HSIL, as shown in the ATHENA trial [9], countering and improving a possible lower specificity of DNA-HPV testing in young people. The protocol proposed will evaluate the performance of CCSP-HPV in women aged 25-29 years compared to older women.

Health managers agreed with the strategy to have one test available for screening and to simplify the flowchart and decided to eliminate cervical cytology from primary health clinics after the introduction of the DNA-HPV test. This is the main decision to prevent the possibility of health professionals performing both HPV-DNA tests and cytology for screening purposes, which would be a bias for cost analysis.

The CCSP-HPV program started in October 2017, and DNA-HPV testing was introduced at the same time in all primary health care units from SUS. After 12 months, 7362 DNA-HPV tests were performed, 99.1% in women within the target age group (25-64 years), well above the planned five thousand tests per year.

Now, it is possible to move forward, and the next steps are pretest of the cost-effectiveness model built in a real scenario, analyze the coverage results achieved after two years, the prevalence of positive tests by HPV type and age group, outcome according to the program flowchart, and the first interim analyses of the cost-effectiveness of the program.

Moving from opportunistic to organized screening has been a challenge, including for the most developed countries. This fact could be partially attributed to the habit and culture of professionals and women who adopted annual testing with cytological examination as the standard to be followed, despite the recommendations establishing longer intervals. The introduction of HPV testing as a primary screening method can break with this cultural practice based on the annual routine cytological examination, allowing or facilitating the establishment of a new practice, now a five-year interval based on scientific evidence. Avoiding overtesting due to repetition at shorter intervals saves money and provides resources to invest in increasing population coverage without the need for additional investments beyond those for the active search for women who are not compliant with the cervical cancer screening.

## Data Availability

The dataset from this study will be safely stored following the principles of research ethics. Upon completion of the study, data may be made available by the corresponding author upon request with justification. The results of this study will be disseminated through publications in journals, presentation of abstracts in scientific congresses and meetings with public health managers.

## Abbreviations

AIS: Adenocarcinoma in situ
ASC-US: Atypical squamous-cells of undetermined significance
CCS-HPV: Cervical Cancer Screening Program with primary DNA-HPV testing
CIN: Cervical intraepithelial neoplasm
EZT: Excision of the transformation zone
FDA: Food and Drug Administration
hr-HPV: High-risk Human Papillomavirus
HSIL: High-grade squamous intraepithelial lesion
LBC: Liquid-based cytology
LSIL: Low-grade squamous intraepithelial lesion
SUS: Sistema Unico de Saude (Public Health System from Brazil)
